# Does walking the day of total hip arthroplasty speed up functional independence? A non-randomized controlled study

**DOI:** 10.1186/s40945-020-00079-7

**Published:** 2020-04-24

**Authors:** Federico Temporiti, Isabella Draghici, Stefano Fusi, Francesco Traverso, Riccardo Ruggeri, Guido Grappiolo, Roberto Gatti

**Affiliations:** 1 Physiotherapy Unit, Humanitas Clinical and Research Center, Via Manzoni 56 - Rozzano, Milan, Italy; 2 Humanitas University, Pieve Emanuele, Milan, Italy; 3 Hip and Knee Orthopaedic Surgery Department, Humanitas Clinical and Research Center, Rozzano, Milan, Italy

**Keywords:** Arthroplasty replacement hip, Early ambulation, Functional independence, Rehabilitation

## Abstract

**Background:**

Few data address modalities for speeding up functional independence in subjects included in a fast-track approach after total hip arthroplasty (THA). The study aim was to assess short-term effects of mobilization and walking the day of THA (WDS) on independence, pain, function and quality of life.

**Methods:**

Seventy-one patients were allocated in a study (SG: *n* = 36) or control (CG: *n* = 35) groups according to time of surgery and recovery from anesthesia. Patients who recovered lower limbs sensitivity (disappearance of sensation deficits) and motility (MRC scale ≥3 at knee, ankle and great toe extension) by 7.00 p.m. made up the SG, whereas patients who underwent surgery later and recovered from anesthesia after 7.00 p.m. made up the CG. SG underwent WDS, whereas CG performed mobilization and walking the day after surgery starting the same physiotherapy program 1 day later. Patients were evaluated for independence (Functional Independence Measure - FIM), pain (Numeric Rating Scale - NRS), hip function (Harris Hip Score - HHS) and quality of life (EuroQoL-5Dimension - EQ. 5D and EQ. 5D-VAS)the day before surgery, at 3 and 7 days in a hospital setting. Analysis of Covariance with age (SG: mean 60.9, SD 9.0; CG: mean 65.5, SD 8.9) and BMI (SG: mean 27.4, SD 2.8; CG: mean 26.7, SD 2.4) as covariates was used to assess between-group differences over time.

**Results:**

Between-groups differences were observed for FIM total and motor scores (*p* = 0.002, mean difference: 2.1, CI_95_: 0.64, 3.7) and FIM self-care (*p* = 0.01, mean difference: 1.7, CI_95_: 0.41, 3) in favor of SG at 3 days. Between-group differences were found for FIM self-care (*p* = 0.021, mean difference: 1.2, CI_95_: 0.18, 2.1) in favor of SG at 7 days. FIM total and motor scores (*p* <  0.001), FIM self-care (*p* = 0.027) and transfer-locomotion (p <  0.001) and HHS (*p* = 0.032) decreased after surgery followed by improvements in postoperative days (*p* ≤ 0.001). No differences were found for NRS, EQ. 5D and EQ. 5D-VAS.

**Conclusions:**

WDS produces additional benefits in patients’ independence in the first week after THA. Absence of pain aggravation or adverse effects on hip function and quality of life may allow clinicians to recommend WDS to promote discharge with functional independence.

## Background

Total hip arthroplasty (THA) is an effective and definitive treatment for end-stage hip osteoarthritis, able to improve functional status, quality of life (QoL) and relieve pain when conservative treatment fails [1]. The rapid achievement of functional independence represents a goal after THA, allowing for decreasing in hospital stay [2, 3]. Over the last few years, length of stay after THA has decreased from several weeks to a few days, allowing for cost reductions in National Health Services of many countries [4]. In fact, several studies showed that improvements in perioperative care have significantly made early discharge possible without increasing complication rates, mainly through the fast track approach [5]. Fast-track consist of a multimodal and interdisciplinary approach applied to patients undergoing elective surgery. It is characterized by procedures designed to administrate preoperative information and education, to alleviate surgical stress response, to treat pain, to support nutrition and to promote early mobilization and walking [6, 7]. In particular, early mobilization and walking consists of transfers, out-of-bed functional activities such as sit-to-stand and maintenance of standing posture and ambulation as soon as possible after spinal anesthesia washout [7–9]. Early mobilization and walking performed in fast track THA patients produce several benefits such as reduction of deep vein thrombosis, increased patient satisfaction, shorter length of stay and hospitalization cost reduction [10–12]. In particular, satisfactory functional status allowing for continuing rehabilitation in autonomy has been described a few days after THA in patients who underwent early mobilization and walking [13]. Moreover, this approach has been reported to have a key role also in reducing physiological and psychological stress related to surgery [14].

Depending on when surgery is scheduled, early mobilization and walking are administered the day of surgery (WDS) or the day after. WDS showed to speed up the readiness for discharge, clinically determined when patients demonstrated independence [15]. However, despite no increase of adverse events has been reported in patients undergoing WDS, previous studies found functional outcomes similar to traditional rehabilitation pathway [16]. It is worth noting that these studies focused on one-year follow-up results, but less is known about the early postoperative phase, in which a great part of functional recovery takes place [16, 17]. In fact, to date no studies have analyzed through specific outcome measures if WDS speeds up functional independence or has any negative effects such as pain increase or QoL decrease during the first days after surgery [18]. Therefore, the aim of this study was to assess short-term effects of WDS on independence, pain, hip function, QoL in patients admitted to fast-track protocol after THA.

## Methods

### Participants

Patients admitted to our Institute to undergo elective THA were longitudinally observed between November 2016 and June 2017. Inclusion criteria were: unilateral cement-less primary THA, age between 40 and 80 years and eligibility in fast track protocol, which consists of ASA Class ≤2, preoperative hemoglobin > 13.0 g/dl, BMI < 35 and preoperative ambulation without crutches for at least 50 m [8, 19]. Exclusion criteria were: musculoskeletal disorders able to influence functional recovery (i.e. symptomatic knee or contralateral hip osteoarthritis, rheumatoid arthritis, spondylitis, severe osteoporosis or upper limb disorders limiting the use of walking aids), neurological disorders, medical problems conditioning the postoperative rehabilitation program (i.e. history of myocardial infarction, pulmonary embolism, deep venous thrombosis or psychiatric disorders) or diagnosed cognitive impairment. Finally, patients scheduled for surgery due to severe dysplasia (Crowe III and IV), traumatic events (i.e. femur or pelvic fractures), or revision surgery were also excluded.

Functional Independence Measure (FIM) represents the primary outcome, however a Minimal Clinically Importance Difference for patients in the first week after THA has not been described. Therefore, a convenience sample of 71 patients were considered, based on a previous study aimed at investigating functional independence in acute phase in patients undergoing different mobilization protocols after THA [20].

Eligibility in the study was assessed by surgeons during hospital pre-admission visit about 2 weeks before surgery and the ID-number of eligible patients was communicated to assessor and physiotherapist who administered WDS. The study was conducted at the Hip and Knee Orthopedic Surgery Department and at the Rehabilitation Department of Humanitas Clinical and Research Hospital of Rozzano, Milan. All patients signed the informed consent the day of admission in hospital, the study was approved by the Internal Ethical Committee (protocol n. CLF16/01) and all patients’ privacy rights were observed.

### Study design

All participants were operated under spinal anesthesia by three orthopedic surgeons of the same Orthopedic Unit using the same procedure of posterolateral approach. Participants were included in a study (SG; *n* = 36) or control (CG; *n* = 35) groups according to time of surgery during the day and time of recovery from spinal anesthesia. Patients who underwent surgery in the morning and recovered lower limbs sensitivity (disappearance of sensation deficits compared to upper limbs) and motility (MRC scale ≥3 at knee extension, ankle dorsiflexion and great toe extension) by 7.00 p.m. were allocated to SG and performed WDS. Patients who underwent surgery in the afternoon and recovered from spinal anesthesia after 7.00 p.m. were placed in the CG. In case of patients operated in the last part of the morning who recovered from spinal anesthesia after 7.00 p.m., they were allocated in CG.

The day of surgery patients of the SG performed WDS under the supervision of the same designated physiotherapist. They were asked to sit with legs out of bed, stand up for a minute and walk twice with walker or crutches for a distance of 10/15 m. In case of inability to achieve these criteria after allocation, patients were excluded from the study. Otherwise, CG performed mobilization and walking the day after surgery, starting the same standardized physiotherapy program of SG 1 day later. The rehabilitation program consisted of two daily individual sessions of 30 min, aimed at restoring independence in basic activities of daily living and improving lower limb motor recovery through mobility and resistance exercises, balance training and functional training such as getting out of bed, sitting on a chair, weight-bearing walking as tolerated, and stairs climbing with crutches. Three physiotherapists not involved in WDS administered the aforementioned rehabilitation program, which lasted until hospital discharge, usually 10 days after surgery. In accordance with previous studies, no movement restrictions or limitations aimed at preventing dislocation were adopted [21, 22]. Discharge criteria, assessed by a nurse and a physiotherapist, were: ability to walk for at least 100 m and climb stairs with crutches, dress the upper part of the body and go to the toilet independently. Moreover, wound had to be dry, hemoglobin > 8.0 g/dl was required and no reporting of dizziness or nausea by patients.

### Outcome measures

All patients were evaluated by a physiotherapist not involved in previous treatments the day before surgery (T0), at three (T1) and seven (T2) days after THA, always at the same time of day. Patients were assessed with total, motor and single subscales (self-care, transfer-locomotion, mobility, bladder and bowel management, cognitive abilities) of the Functional Independence Measure (FIM), which represents a 18-item clinician-reported scale ranging from 18 to 126 (maximum independence) to evaluate independence during basic daily activities [23, 24]. Pain was assessed after got out of bed and set on a chair in autonomy using the Numeric Rating Scale (NRS). It consists of 11-point numeric scale with a score ranging from 0 to 10 (maximum pain) to evaluate pain intensity after THA [25]. Hip function was assessed using Harris Hip Score (HHS), which is a valid tool ranging from 0 to 100 (maximum function) to assess hip functional status especially after surgery [26]. Quality of life was assessed in hospital using EQ. 5D (5-Levels), which consists of two tools: EQ. 5D descriptive system and EQ. 5D-VAS [17]. The EQ. 5D descriptive system is a questionnaire evaluating 5 dimensions of health (mobility, self-care, usual activities, pain/discomfort and anxiety/depression) from which a score ranging from 0 to 1 (maximum health status) is derived. The EQ. 5D-VAS is a visual analogue scale ranging from 0 to 100 (maximum health status) where patients has to indicate the level of health status. Finally, demographic variables such as age and BMI were also collected at baseline due to their strong association with functional outcomes after THA [27]. Standardized instructions were given to patients to explain the assessment tools before each evaluation session.

### Statistical analysis

Data were described as mean and standard deviation and were analyzed using SPSS Statistics 20.0. All measurements were checked for normality through Shapiro-Wilk test, whereas Levene’s test was used to assess equality of variances across the two groups. Subsequently, t-test for independent samples or chi-square test were used to assess between-group differences in terms of age, gender, height, weight, BMI and outcome measures at baseline. Analysis of Covariance (ANCOVA) with Time as within-subjects variable and Group as the between-subjects variable was used to assess between-group differences over time. Age and Body Mass Index (BMI) were included in the analysis as covariates [27]. In case of significance, Univariate ANCOVA was used to compare between-group values at each time-point, whereas One-way repeated measure ANCOVA was used to perform within-group comparison between T0, T1 and T2. Post-hoc analysis with Bonferroni correction was performed in case of significance of One-way repeated measure ANCOVA and adjusted *p*-value was reported. In addition, two-tails t-test was used to compare between-group differences of T2-T1 (delta). Finally, the effect size and 95% confidence interval between the two groups was quantified at each time-point using Hedges’ *g*. Effect size was considered small (0.2), medium (0.5) or large (≥0.8) [28]. The statistical level of significance was set at α = 0.05.

## Results

No patients withdrew from the study and all patients of the SG reached the criteria for WDS. One patient in each group was excluded from analysis due to headache and hypertension, which influenced their mobilization during the first postoperative day. Until the seventh postoperative day, all other patients performed the rehabilitative program consisting of 14 sessions (including WDS) for SG and 13 sessions for CG. No adverse events occurred. Both groups were homogeneous for gender, body height and weight, BMI and outcome measures at baseline, whereas a difference was observed for age (SG: mean 60.9, SD 9.0 years; CG: mean 65.5, SD 8.9 years; *p* = 0.03) (Table 1).

**Table 1:** Baseline characteristics of study and control groups. Adjusted data by age and BMI are shown as mean and SD

	T0		
	SG	CG	p-value
**Age**	60.9 ± 9	65.5 ± 8.9	**0.03**
**Gender**	15 M/20 W	13 M/21 W	0.116
**Height**	1.71 ± 0.08	1.72 ± 0.09	0.628
**Weight**	81.1 ± 14.1	79.6 ± 11.5	0.637
**BMI**	27.4 ± 2.8	26.7 ± 2.4	0.249
**FIM-TOT**	115.1 ± 3.4	114.4 ± 3.3	0.372
**FIM-MOT**	80.1 ± 3.4	79.4 ± 3.3	0.372
**FIM-SC**	39.3 ± 2.2	38.7 ± 2.2	0.184
**FIM-SP**	14 ± 0	14 ± 0	1
**FIM-TL**	26.8 ± 1.6	26.7 ± 1.9	0.732
**FIM-C**	35 ± 0	35 ± 0	1
**NRS**	3.7 ± 2.4	3.4 ± 2.7	0.572
**HHS**	62.4 ± 14.4	60.7 ± 13.9	0.54
**EQ 5D**	0.4 ± 0.2	0.4 ± 0.2	0.49
**EQ 5D-VAS**	56 ± 17.5	55.9 ± 15.8	0.854

*SG* study group, *CG* control group, *T0* time-point 0, *FIM-TOT* FIM total score, *FIM-MOT* FIM motor score, *FIM-SC* FIM self-care, *FIM-SP* FIM sphinteric control, *FIM-TL* FIM transfer-locomotion, *FIM-C* FIM cognitive, *NRS* numeric rating scale, *HHS* Harris Hip Score, *EQ. 5D* EuroQoL-5Dimension, *EQ. 5D-VAS*: EuroQoL-5Dimension Visual Analogue Scale

Table 2 shows between-group differences over time adjusted for age and BMI. A Group effect was found for FIM total score (FIM-TOT), FIM motor score (FIM-MOT) and FIM subscale related to self-care (FIM-SC) in favor of SG. An effect of Time was found for FIM-TOT, FIM-MOT, FIM-SC, FIM subscale related to transfer and locomotion (FIM-TL) and HHS. No significant differences were found for EQ. 5D and EQ. 5D-VAS (Table 2).

**Table 2:** Outcome measures before surgery (T0), after three (T1) and 7 days (T2) in study and control groups adjusted by age and BMI. Data are shown as mean and SD

	SG			CG			*p*-value Time Factor	*p*-value Group Factor	*p*-value Time x Group Interaction
	T0	T1	T2	T0	T1	T2			
**FIM-TOT**	114.9 ± 3.4	102.5 ± 3.1	104.9 ± 2.9	114.5 ± 3.4	100.4 ± 3.1	103.7 ± 2.9	**< 0.001**	**0.042**	0.120
**FIM-MOT**	79.9 ± 3.4	67.5 ± 3.1	69.9 ± 2.9	79.5 ± 3.4	65.4 ± 3.1	68.7 ± 2.9	**< 0.001**	**0.042**	0.120
**FIM-SC**	39.3 ± 2.2	33.5 ± 2.6	35.1 ± 2	38.7 ± 2.2	31.8 ± 2.6	34 ± 1.9	**0.027**	**0.01**	0.199
**FIM-SP**	14 ± 0	14 ± 0	14 ± 0	14 ± 0	14 ± 0	14 ± 0	–	–	–
**FIM-TL**	26.7 ± 1.7	20 ± 1.1	20.7 ± 1.7	26.8 ± 1.7	19.5 ± 1.1	20.7 ± 1.7	**< 0.001**	0.669	0.441
**FIM-C**	35 ± 0	35 ± 0	35 ± 0	35 ± 0	35 ± 0	35 ± 0	–	–	–
**NRS**	3.7 ± 2.6	3.8 ± 2.1	3.6 ± 2.1	3.3 ± 2.6	3.9 ± 2.1	3.4 ± 2.1	0.894	0.709	0.788
**HHS**	61.5 ± 13.9	49.5 ± 8.1	51.4 ± 8.8	61.6 ± 14.2	48.5 ± 8.1	49.9 ± 8.8	**0.032**	0.672	0.882
**EQ 5D**	0.39 ± 0.2	0.51 ± 0.15	0.53 ± 0.12	0.38 ± 0.2	0.53 ± 0.14	0.54 ± 0.13	0.660	0.916	0.557
**EQ 5D-VAS**	55.7 ± 17.1	64.4 ± 16.9	69.5 ± 16.6	56.2 ± 17.1	60.2 ± 16.9	64.8 ± 16.6	0.361	0.458	0.157

*SG* study group, *CG* control group, *T0* time-point 0, *T1* time-point 1, *T2* time-point 2, *FIM-TOT* FIM total score, *FIM-MOT* FIM motor score, *FIM-SC* FIM self-care, *FIM-SP* FIM sphinteric control, *FIM-TL* FIM transfer-locomotion, *FIM-C* FIM cognitive, *NRS* Numeric Rating Scale, *HHS* Harris Hip Score, *EQ. 5D* EuroQoL-5Dimension, *EQ. 5D-VAS* EuroQoL-5Dimension Visual Analogue Scale

At 3 days, between-group post-hoc analysis revealed significant differences for FIM-TOT and FIM-MOT (*p* = 0.006, mean difference: 2.1, CI_95_: 0.64, 3.7, Hedges’ *g*: 0.67, CI_95_: 0.2, 1.15) and for FIM-SC (*p* = 0.01, mean difference: 1.7, CI_95_: 0.41, 3, Hedges’ *g*: 0.67, CI_95_: 0.17, 1.13) in favor of SG. At 7 days, differences for FIM-SC (*p* = 0.021, mean difference: 1.2, CI_95_: 0.18, 2.1, Hedges’ *g*: 0.56, CI_95_: 0.09, 1.04) remained significant in favor of SG.

As expected considering surgery, within-group post-hoc analysis comparing baseline with third and the seventh postoperative days showed significant decrease for FIM-TOT, FIM-MOT, FIM-SC, FIM-TL and HHS (*P* ≤ 0.001) in both groups. Comparison between deltas (T2-T1) showed significant differences for FIM-TOT and FIM-MOT in favor of CG (SG: mean 2.3, SD 1.8; CG: mean 3.4, SD 2.5; *p* = 0.042). In fact, CG revealed FIM-TOT and FIM-MOT scores at 7 days (mean 103.7, SD 2.9 and mean 68.7, SD 2.9) similar to that obtained by SG at 3 days (mean 102.5, SD 3.1 and mean 67.5, SD 3.1).

## Discussion

The aim of the study was to assess short-term effects of WDS on independence, pain, hip function and quality of life in patients admitted to fast track protocol for THA. At 3 days, patients who underwent WDS revealed independence similar to patients of the control group at 7 days. Moreover, absence of pain aggravation or any adverse effect on hip function and QoL may allow clinicians to recommend this approach when an early recovery of functional independence is expected. At baseline, no significant between-group differences were found except for age, which was slightly lower in SG. This characteristic might not have influenced results, since age has been taken into account in our statistical model and other features are considered as stronger predictive outcome factors, such as pre-operative status, comorbidities and anxiety and depression [18]. A study considered age an influencing factor of health-related QoL after THA, but the threshold of this prognostic factor is age greater than 70 years old, greater than age of our study participants [15].

As expected considering surgery, patients showed significant decrease in functional independence immediately after surgery, followed by improvements during the postoperative period, but this level remained lower than baseline at 7 days. However, this kind of surgery does not produce an important loss of independence and consequently between-group differences for FIM total and motor scores in favor of SG cannot be expected to be large. In addition, Minimal Clinically Importance Difference for FIM in patients in first week after THA has not been described and consequently, impacts of our results in terms of clinical relevance cannot be fully understood. The presented results may be related to fear of movement affecting patients after THA. In fact, Olsson described high Tampa Scale of Kinesiophobia score in patients scheduled for THA, which represents a feeling of fear toward pain affecting rehabilitation and reducing functional recovery speed [29]. Consequently, fear of movement reduction represents an interdisciplinary goal in patients who undergo THA [28]. Moreover, awareness to have improved health status and that pain was not a limiting factor for mobilization might have also contributed to enhance patients’ functional independence after WDS [30, 31]. In fact, the approach adopted in SG might have increased the use of affected limb in functional activies, reducing adaptive stategies with the non-affected limb and functional dependence on healthcare personnel and caregivers [32]. Finally, we cannot exclude that these differences, might also depend on the additional number of rehabilitative sessions performed by SG (6 sessions in SG versus 5 sessions in CG at 3 days). In fact, it is worth noting that from third to seventh postoperative day, CG showed a recovery of independence similar to those reached by SG in the first 3 days. However, at seventh postoperative day FIM-SC score was still greater in SG patients. Our results suggest that WDS could play a role in promoting discharge with functional independence after THA, especially when patients are discharged at home without the presence of a caregiver.

Pain represents the main affecting factor on functional recovery after THA [33]. In this study, no between-group differences were observed for pain, which was similar to that reported before surgery. Moreover, since pain score was lower than 5 points (moderate pain) during mobilization, it has been reported as too low to influence daily activities [17].

The study showed a significant decrease in hip function in both groups from baseline to third and seventh postoperative days due to surgical procedure. As expected, this decrease was greater than Minimal Clinically Important Difference (4 points) for HHS estimated for this condition [34]. Moreover, based on traditional categorization, hip function of our patients can be considered still poor (HHS < 70) at 7 days after surgery [35]. Our findings are consistent with literature, since studies reported hip function decrease immediately after THA, followed by achievement of preoperative values during the first postoperative month [36].

Quality of life is expected to improve within 6 months after THA, with no differences between patients admitted to fast track setup or conventional perioperative care regime [37]. In the current study, no between-group differences were found for QoL, which, despite the positive trend, revealed also no significant improvements from baseline. However, at three and 7 days, EQ. 5D of our patients was slightly higher than the score reported 2–3 days after surgery by other studies focused on patients’ quality of life after THA [17]. This positive trend might depend on patient-centered care model, typical of fast-track setting [38]. Moreover, since EQ. 5D was collected in hospital, the rapid gain in functional independence and absence of postoperative complication in both groups could have played a role in determining these results. Despite promising results, several limitations of the present study need to be underlined. First, being a non-randomized controlled study, patients were allocated to study or control group according to recovery time from spinal anesthesia. Consequently, we cannot exclude that higher dose of anesthesia may have been administered to more compromised patients, determining a longer washout and allocation in CG. However, no significant differences were found for hip function at baseline and no adverse intraoperative events occurred. Moreover, it is worth noting that all patients were operated by the same orthopedic team and were under the care of the same working team. Second, the current study design is characterized by lack of a-priori sample size analysis and results may be unpowered or the effect of the WDS may be underestimated. Third, two groups were not comparable in terms of age. However, although previous studies reported that this difference should not have influenced our findings, age was included in statistical model as covariate [15]. In addition, the number of comorbidities and mental disorders can be considered influencing factors for functional recovery after THA and the lack of data regarding their presence must be acknowledged as a limitation [18, 27]. Four, the perioperative assessment of quality of life through EQ. 5D and EQ. 5D-VAS was performed in hospital and caution is needed to generalize results. Moreover, pain at rest or during WDS was not assessed, but it could have provided a better sample characterization. Finally, we did not assess the amount of kinesiophobia or other influencing factors through administration of specific tools, which could help to better explain the difference observed in patients’ functional independence.

## Conclusion

Results of this study showed that WDS speeds up recovery of functional independence after THA without adverse effects on pain, hip function and quality of life. WDS could play a role in promoting discharge with functional independence especially in patients discharged at home without the presence of caregivers. In fact, speeding up functional independence may be useful in the current scenario where length of stay is being decreased from several weeks to a few days. Future studies are needed to explore the role of WDS on kinesiophobia or other factors limiting functional independence in the first days after THA.

## Data Availability

The dataset of the current study is available from the corresponding author.
